# Experimental Study on Distribution of Landslide Thrust in Pile-Anchor Structure based on Photoelastic Technique

**DOI:** 10.3390/ma13061358

**Published:** 2020-03-17

**Authors:** Xunchang Li, Rui Xu, Wei Yang, Pingan Li, Ke Yang, Wenyong Zhang

**Affiliations:** School of Geology Engineering and Geomatics, Chang’an University, Xi’an 710054, China; dcdgx12@chd.edu.cn (X.L.); yw2014@chd.edu.cn (W.Y.); li7706195@163.com (P.L.); yangke9702@163.com (K.Y.); 2019126127@chd.edu.cn (W.Z.)

**Keywords:** pile-anchor support system, landslide thrust, load sharing ratio, photoelastic test, anchor tension, internal force distribution

## Abstract

This paper aimed to perform systematical study on the distribution of landslide thrust in pile-anchor support system, which has been a widely applicable treatment method in landslide control with safety, highly efficiency and adaptation. The advantage of photoelastic technique is visualization of strain and stress fields, therefore photoelastic model tests are conducted to show the distribution of landslide thrust in pile-anchor structure before failure in landslide. The effects of different materials and pile lengths are investigated by 6 photoelastic test cases under different loading conditions. It can be found from quantitative analysis of experimental results that load proportion of anchor would increase gradually with the decrease of pile embedded depth or the increase of landslide thrust force. Meanwhile, landslide thrust distribution in pile-anchor structure is directly affected by the stiffness of piles. The pile-anchor structure is significantly better at reducing bending moment value and optimizing bending moment distribution of pile. Finally, some theoretical analysis and design suggestions are proposed based on the experimental study.

## 1. Introduction

In recent years, human engineering activity has become one of the main challenges on natural environment [1]. First in the firing-line has been a series of geological hazards, especially landslides. Landslides induced by human engineering activity and local environment, such as seasonal heavy rainfall, have been a serious problem all around world [2,3,4,5]. The anti-sliding pile is a widely applicable treatment method in landslide control with safety, highly efficiency and adaptation [6]. The anti-sliding pile can transfer body and shear forces from the landslide mass to underlying stable layers in order to stop landslide from moving [7,8]. While anti-slide pile is the preferred method of controlling landslide in mountainous regions, when it comes to the large scale of landslides, sometimes a traditional cantilever single row pile can hardly provide considerable resistance to landslides [9,10], and if forced to be chosen (for example, larger pile diameter or longer pile length), the cost would be immense [3]. To such a situation, pile-anchor support system, as a new sliding bracing structure, has been successfully used to control large scale landslides, because of many advantages, such as more reasonable distribution of stress, lesser slope deformation, more anti-sliding force ability, more stably and easily constructed.

Recently, some researchers focus on the relationships between slope stability and pile-anchor support system worldwide, including the study in the mountain region of China [3,11]. However, the mechanism of the pile-anchor support system in landslides is still needed to be systematically investigated [11]. Moreover, the distribution of sliding thrust forces, especially load sharing ratio of landslide thrust between anchor and pile in landslides is a key factor in the theory and design, but only a few scholars have conducted researches on landslide thrust distribution in a pile-anchor support system. These studies mainly focused on the monitoring data of the pile-anchor support system in the field tests, model tests and engineering projects [12,13,14]. However, it is noteworthy that most of the experimental studies carried out previously in this context focused on the specification of macroscopic mechanical responses of aggregated geo-materials and support structure rather than their interior stress fields [12]. It is almost impossible to reveal the force network distribution in geo-materials and support structure by using the traditional geotechnical experimental methods. Hence, an effective experimental method to study the mechanics of the pile-anchor structure and allow for visualization of the force information at the materials would be helpful to understand the mechanism of the pile-anchor support system [11].

With regard to stress visualization, the distribution characteristics of isochromatic fringe of strain and stress can be successfully observed based on the refringence in optically materials by photoelastic technique, and then the strain and stress fields can be also quantitatively analyzed [13,14,15,16,17,18]. The force details of the materials can be calculated based on the photoelastic response of the materials as viewed in polarized light field, while recording useful additional information in the normal light field [19]. Recently, the photoelastic experimental method has been extensively applied to the geotechnical mechanical research field [18]. Important phenomena have been reported based on this experimental method [20]. 

In this paper, in order to understand the load sharing ratio and distribution of landslide thrust in pile-anchor structure before failure in landslide, the physical scale model tests are conducted using photoelastic technique. The effects of different material and pile length are investigated by 6 photoelastic test cases under different loading conditions. Based on the photoelastic response images of the pile-anchor support system, the photoelastic jamming theory is also applied to explain the characteristic of stress distribution, and then the law of distribution of landslide thrust in pile-anchor support system, as landslide thrust increases. At last, some design suggestions are proposed based on the experimental study. This research is expected to improve understanding of the mechanism of the pile-anchor structure.

## 2. Methodology

Photoelastic technique has significant advantages in displacement, strain and stress tracking, while the photoelastic method can directly present the force network information and the contact force of each part in the pile-anchor support system which could be applied to the pile-anchor support system research.

### 2.1. The Prototype Landslide and the Law of Similitude

**Figure 1:**
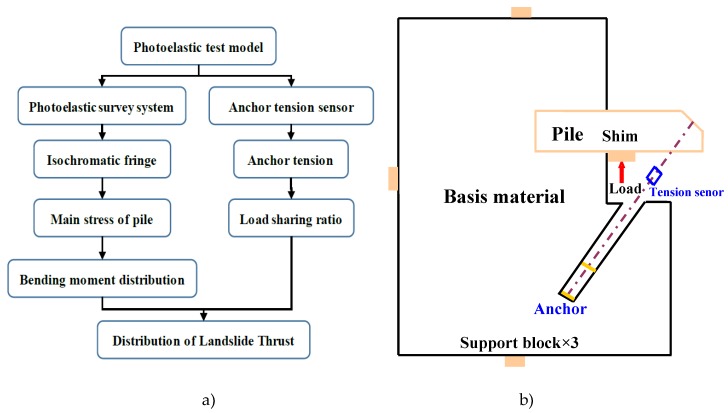
Experimental setup. (**a**) Flow chart of test, (**b**) schematic of physical landslide model.

**Figure 2:**
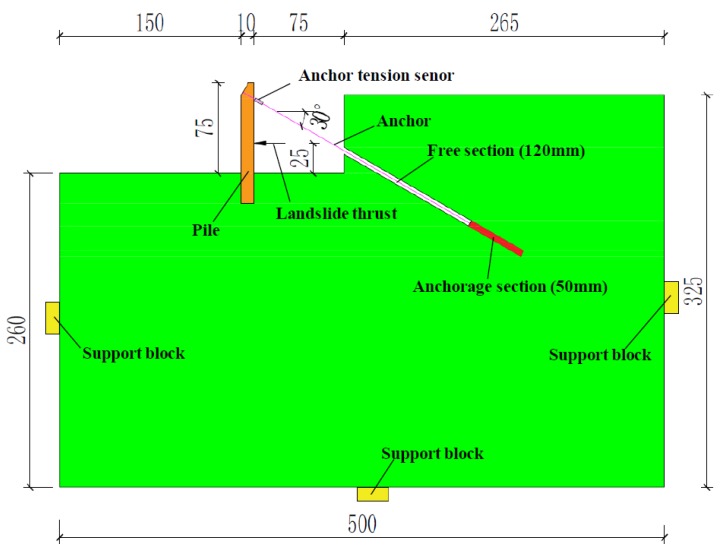
Geometry of physical landslide model (Unit: mm).

The photoelastic test model was designed based on a typical homogeneous landslide, which was supported by a pile-anchor support system, shown in Figure 1. According to the dimensions and capacity of the photoelastic experimental apparatus, the similarity ratio *λ_L_* of geometric size was determined to be 1:100. The size of the pile-anchor support system is shown in Figure 2.

The model is 500 mm in length, 325 mm in height and 5.86 mm in thickness, as shown in Figure 1. The pile-anchor structure was placed in the landslide model. The section size and length of the pile is 8 × 10 mm and 100~150 mm (including 3 cases: 150 mm, 125 mm and 100 mm), respectively. The geometrical conditions of pile are listed in Table 1.

### 2.2. Model Preparation and Installation

The landslide soil used in the test was epoxy resin photoelastic material. In this work, two kinds of typical pile (epoxy resin and aluminum alloy, respectively) were made to study how different stiffness of pile affect the stress distribution characteristics of the pile-anchor structure. The elastic modulus of epoxy resin (*E_H_*) and aluminum alloy (*E_L_*) is about 3.5 *GPa* and 70 *GPa*, respectively. The anchor was made of brass wire, taking epoxy resin as anchorage.

In order to reduce the influence of assembling stress and initial stress, the test pile and anchor were fixed to the predesigned positon in photoelastic test model after stress relief.

### 2.3. Loading Scheme

**Figure 3:**
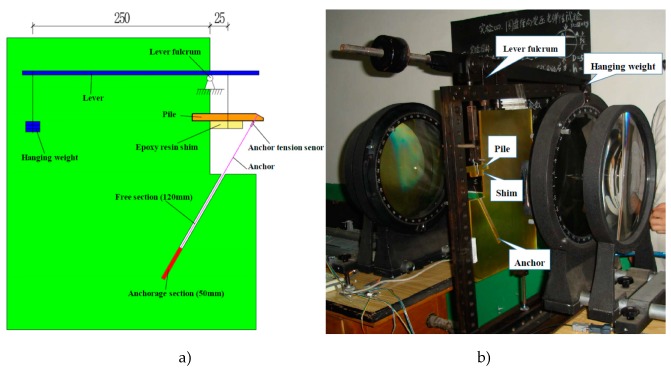
Loading design structure. (**a**) Schematic of loading design structure, (**b**) physical photo of loading design structure.

The effects of different material and pile length are investigated by 6 photoelastic test cases (2 kinds of pile material and 3 kinds of pile length) under different loading conditions. According to the lever principle, multistage loading was adopted using hanging weight, and the ratio of leverage is 1:10, as shown in Figure 3. Based on the theoretical hypothesis of triangular distribution of lateral force to the cantilever pile, the loading location was set in the 1/3 cantilever segment of pile above sliding surface. In order to avoid the stress concentration in load location, an epoxy resin shim was set on the loading location.

By multi-stage loading, the hanging weight is gradually increased from 5 kg to 25 kg, with 5 kg per stage. According to leverage ratio shown in Figure 3, the corresponding values of landslide thrust are 49 N, 98 N, 147 N, 196 N and 245 N. 

### 2.4. Measuring Scheme

#### 2.4.1. Anchor Tension Sensor

**Figure 4:**
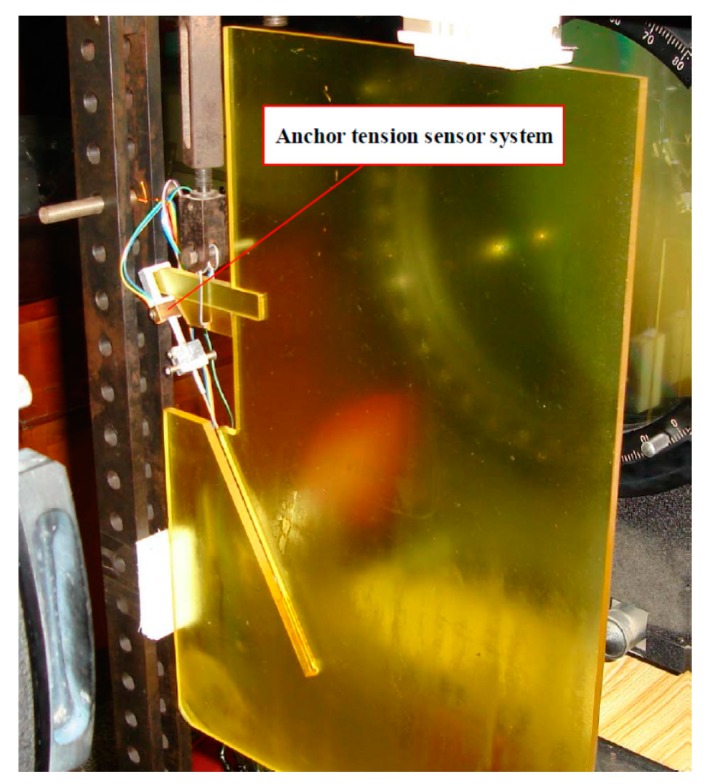
Anchor tension sensor.

In order to obtain the tensile stress of the anchor, an anchor tension sensor was designed for the test. The main part of this anchor tension sensor is a steel gasket with a strain gauge bonded. The anchor tension sensor is connected in series on the connection between the anchor and the joint point at the end of the pile. When the load is borne on the anti-slide pile body, the anchor shares the corresponding load accordingly, which is tested by the anchor tension sensor (Xi’an Jiaotong University. Xi’an, China) and stored in the test system, as shown in Figure 4.

Before the test, the tension sensor was first calibrated with the test system. The calibrated standard force was provided by the electronic universal testing machine (KYKY Technology Co. Beijing, China). It could be seen that the output result of the anchor tension sensor is well correlated. Moreover, the strain varied almost linearly with the calibration load. This linear equation is expressed as follows:

*P* = *0.374x* − *17.892* (1)

where *x* is the measured strain value on anchor head, *P* is the tension value corresponding to the strain with unit of *N*.

#### 2.4.2. Photoelastic Survey System

The test system includes two parts: photoelastic experimental stress testing system and anchor tension test system. During multi-stage loading process by the lever structure, the anchor tension test system collects and stores the load data in real time through a computer. 

In this study, the effects of different material and pile length are investigated by 6 photoelastic test cases under multi-stage loading. The corresponding values of multi-stage loading are 49 N, 98 N, 147 N, 196 N and 245 N.

In this test, the load exemplary isochromatic fringe in photoelastic experiment is visualized and recorded. According to the experimental calibration and statistics before the test, the main stress *σ_1_* of each point at the edge of pile body can be counted by calibration Equation (2).

*σ_1_* = *f* × *m* / *b* (2)

where *f* is the stress fringe value of the material, usually measured with the same material before the test. *m* is the isochromatic fringe order. *b* is the thickness of the model (5.86 mm in this test).

According to the mechanics of materials, with the main stress *σ_1_*, the bending moment value *M* of each point at the edge of pile body can be obtained from Equation (3).

*M* = (*σ_1f_* − *σ_1b_*) *W_z_* (3)

where *σ_1f_* is the main stress *σ_1_* in the front of the pile. *σ_1b_* is the main stress *σ_1_* in the back of the pile. *W_z_* is the section modulus in bending of the pile.

## 3. Results and Discussion

### 3.1. Isochromatic Fringe Phenomena and Anchor Tension

**Figure 5:**
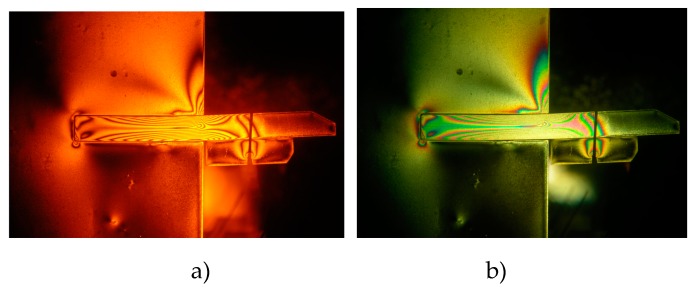
Typical isochromatic fringe of epoxy resin pile (No.1). (**a**) Isochromatic fringe under condition of monochromater, (**b**) isochromatic fringe under condition of white light.

Distribution characteristics of isochromatic fringe are the main test contents of photoelastic experiment. The load exemplary isochromatic fringe in photoelastic experiment is visualized in Figure 5.

The load value and distribution can be obtained by analysis of isochromatic fringe based on stress-optical law. At last, based on the results of comprehensive analysis of the data from anchor tension sensor and isochromatic fringe, the tension of anchor and the ratio of loading share between anchor and pile can be obtained directly, as listed in Table 2 (epoxy resin pile model) and Table 3 (aluminum alloy pile model).

### 3.2. Landslide Thrust Share in the Pile-Anchor Support System

**Figure 6:**
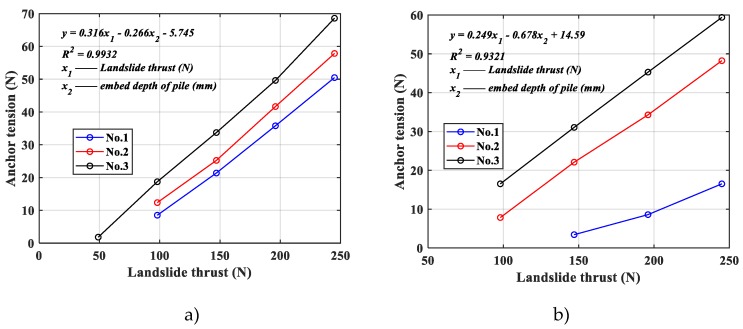
Comparison of anchor tension. (**a**) Anchor tension in epoxy resin pile model, (**b**) anchor tension in aluminum alloy pile model.

The main goal of current research on the load sharing ratio of landslide thrust in the pile-anchor support system is to develop structural calculation model and to propose reasonable design method. However, it is necessary to first understand the influence rule of different design parameters on the pile-anchor support system. The first thing to consider here is the embed depth of pile. The change in anchor tension for different embed depths of pile is shown in Figure 6. 

Generally speaking, anchor tension would increase with the increase of landslide thrust, as shown in Figure 6. Moreover, anchor tension relative to landslide thrust in same pile material under different embed depth of pile, then, grows basically in a straight line. It can be seen from Figure 6 that the slope of all of these straight lines is nearly same regardless of what the embed depth of pile is. The relationship between anchor tension, landslide thrust and embed depth of pile, is linear (R^2^>0.93) by statistics, as the formula shown in Figure 6. 

### 3.3. Load Sharing Ratio of Anchor

**Figure 7:**
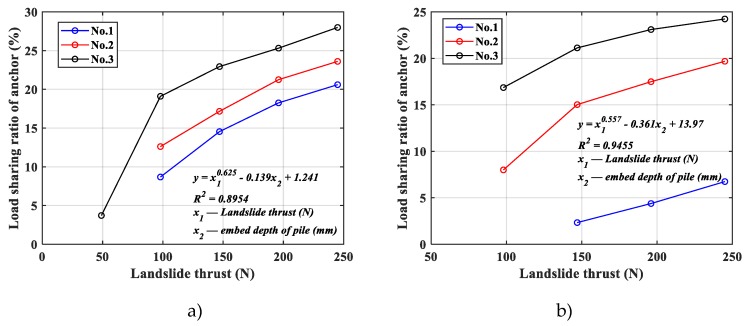
Load sharing ratio of anchor. (**a**) Ratio in epoxy resin pile model, (**b**) ratio in aluminum alloy pile model.

It can be found from quantitative analysis of experimental results that the horizontal loading on the pile would also increase as anchor tension with the increase of landslide thrust force, and the characteristics and linear functional relationship of horizontal loading on the pile was nearly the same as that of anchor tension. Therefore, more importantly, it is necessary to first understand the character of the ratio of loading share of anchor on pile-anchor support system. The change in the load sharing ratio of anchor for different pile anchorage depth is shown in Figure 7.

As the Figure 7 shows, the slope of all of these exponential curve is nearly same, and the load sharing ratio of anchor increased proportionally with a decrease of embed depth of pile and an increase of landslide thrust. Obviously, the decrease of embed depth of pile would decrease the capacity for work of pile in pile-anchor support system, so the load sharing ratio of anchor would increase proportionally. On the other words, various reasons contribute to the relative increase of load sharing ratio of anchor, with embed depth, size and material of pile believed to be the primary ones, which could collectively be called ‘global rigidity’ of pile. The decrease of ‘global rigidity’ of pile would increase the load sharing ratio of anchor.

### 3.4. Effect of Increased Landslide Thrust

Another point worth noting is the relation between the load sharing ratio of anchor and the landslide thrust. Firstly, with the increase of landslide thrust, the anchor tension and the load sharing ratio of anchor would increase. As shown in Figure 7, measured by load shares, the bigger the landslide thrust is, the smaller the load sharing ratio of pile is, the bigger the load sharing ratio of anchor is. 

Secondly, different from linear function relation between the anchor tension and the landslide thrust, there is an obvious exponential function relation between the load sharing ratio of anchor and the landslide thrust. This led that the rate increase of the load sharing ratio of anchor would be smaller than that of the landslide thrust. It is found that the load sharing ratio of anchor showed an increasing trend but a slower and slower upward trend with the increase of landslide thrust, especially when the load sharing ratio of anchor is more than 20%. On current trend of the exponential curve in Figure 7, it is projected to end up at or close to the 30% level in the test of No.3 pile. In other words, as landslide thrust increases, the change of the load sharing ratio between anchor and pile would be slower and slower, and the load sharing ratio between anchor and pile would be finally expected to remain roughly stable. As mentioned above, the ultimate stable state of the load sharing ratio is relevant to the ratio of the ‘global rigidity’ between anchor and pile in the pile-anchor support system. Therefore, as shown in Figure 7, different embed depth of pile would lead to different ultimate stable states of the load sharing ratio.

### 3.5. Effect of Pile Material

**Figure 8:**
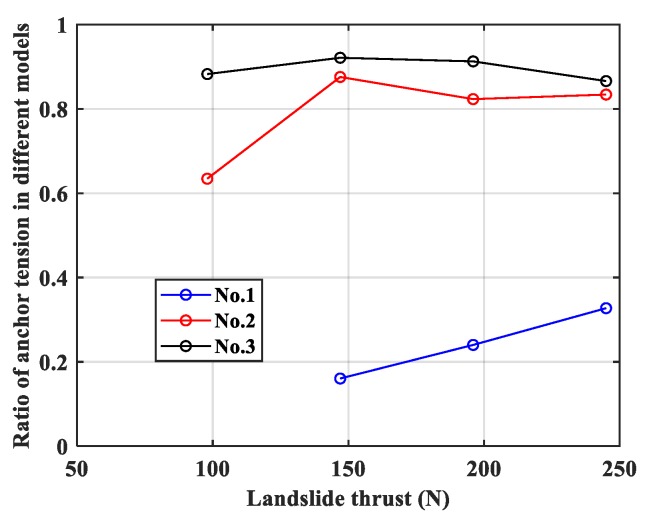
Ratio of anchor tension in different pile material models.

As above statistical analysis showed, the ratio of the ‘global rigidity’ between anchor and pile in the pile-anchor support system would directly affect the load sharing ratio of landslide thrust. In addition to embed depth of pile, pile material also has a great effect on bearing landslide thrust. Ratio of anchor tension in aluminum alloy pile model to in epoxy resin pile model was shown in Figure 8. 

The rigidity of the aluminum alloy material is about 20 times that of the epoxy resin material, therefore the anchor tension in epoxy resin pile model was obviously more than that in the aluminum alloy pile model. As shown in Figure 8, when the embed depth of pile is small, the ratio of anchor tension in different models is closer to 1, showing that pile material has a lesser effect on bearing landslide thrust in this case. The proportion of landslide thrust is higher only in the case of larger embedding depth.

Considering the practical engineering, there are two ways to increase the stiffness of pile. One way is to increase the cross-sectional area of the pile body. Another way is to increase the elastic modulus of pile body, which can be achieved by strengthening and using high grade of steel concrete. However, with no exception of the two methods can create engineering investment increased dramatically. It can be seen from the above analysis that the increase of stiffness for short pile has made little visible difference to load share of the landslide thrust. Moreover, in the case of the pile-anchor support system in actual engineering, the embed depth of pile is generally shorter than that in ordinary anti-slide pile. By the increase of the stiffness of pile, the effect of such measures seems limited, given its investment proportion.

### 3.6. Influence of Anchor on the Internal Force Distribution of Pile

On the basis of the above study on the load sharing ratio of landslide thrust, further research should be made into the problem of internal force distribution of pile. Photoelastic technique is an effective experimental method to study the mechanics of the pile-anchor structure and allow for visualization of the force information at the materials, would be helpful to understand the mechanism of the pile-anchor support system. For this purpose, comparison tests have been conducted based on photoelastic technique. 

**Figure 9:**
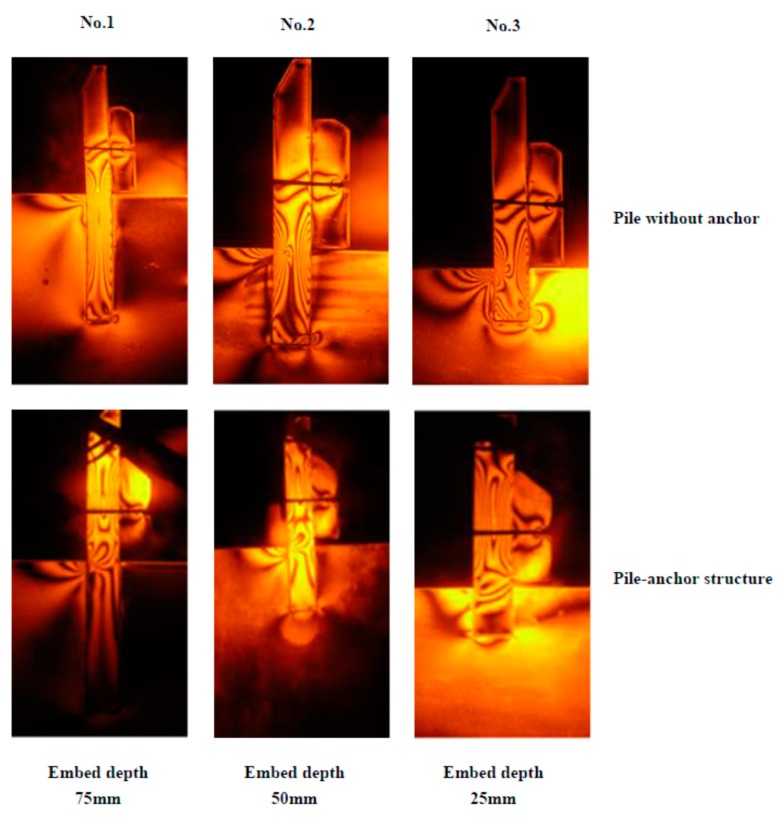
Isochromatic fringe of epoxy resin pile.

In this comparison test, both of the landslide soil and pile used in the test were epoxy resin photoelastic material. The geometrical conditions of pile are listed in Table 1. The hanging weight is 25 kg, the corresponding values of landslide thrust are 245 N. Moreover, for comparison, an anti-slide pile without anchor test has been also done under the same conditions, and all the test-range of pile is conformed from 20 mm under sliding surface to 50 mm above sliding surface. The load exemplary isochromatic fringe in photoelastic experiment is visualized in Figure 9.

**Figure 10:**
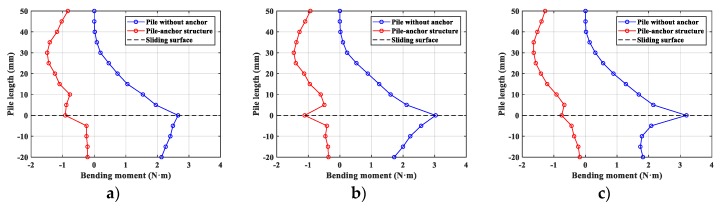
Bending moment distribution of the pile. (**a**) No.1 (embed depth 75 mm), (**b**) No.2 (embed depth 50 mm), (**c**) No.3 (embed depth 25 mm).

**Figure 11:**
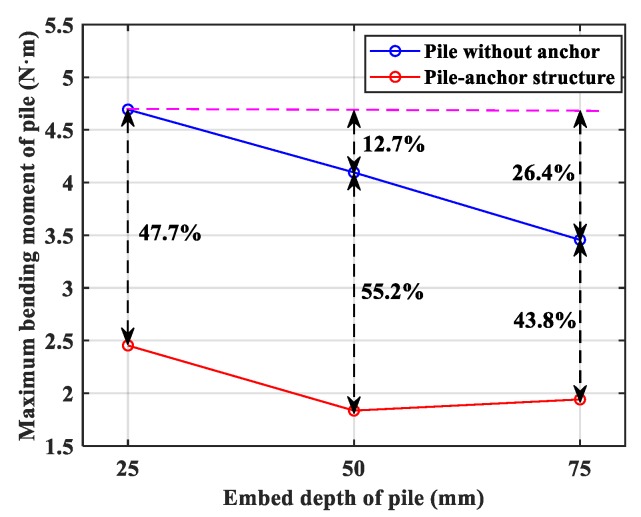
Maximum bending moment of the pile. (The double arrow lines denote the percentage of decrease).

Based on above theoretical formula, the bending moment distribution of each specimen could be obtained by the data analysis on the isochromatic fringe, as shown in Figure 10. Comparing these two different structure systems, the pile-anchor structure is significantly better at reducing bending moment value and optimizing bending moment distribution of pile. Statistically, anchor could respectively decrease the maximum bending moment of pile by 43.8%, 55.2% and 47.7% on No.1, No.2 and No.3 pile, as shown in Figure 11. 

Secondly, it is necessary to understand the influence rule of different embed depth of pile on bending moment distribution of pile. Clearly, in the case of pile without anchor structure, from the comparison of the different embed depth of piles, the corresponding ratio of embed depth of piles is 1:2:3, and the corresponding ratio of maximum bending moment is 1:0.873:0.736, as shown in Figure 11. The ratio of bending moment decrease is obviously less than that of pile embed depth increase. However, anchor can decrease the maximum bending moment of pile by almost 50%. Therefore, it is an injustice to blindly increase the pile length to reduce the internal force on pile, especially considering the low-cost and easy-construction anchor.

Thirdly, anchor is an effective way to optimize bending moment distribution of pile. As the pile without anchor shown in Figure 10, the maximum bending moment of the pile is observed on the sliding surface, and the value is fairly large. Instead, in the pile-anchor structure, the maximum bending moment of the pile is observed at the point that 30 mm (approximately half of the cantilever part of pile) above the sliding surface, and the bending moment distribution is more uniform.

**Figure 12:**
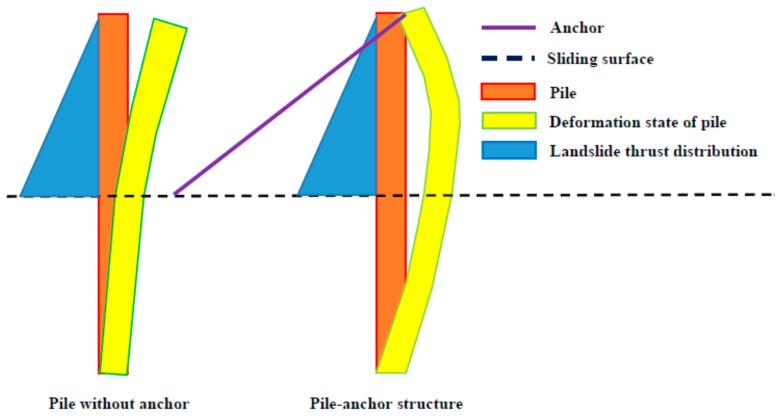
Deformation state of pile in different support structure systems.

In addition, anchor would affect the direction of bending moment distribution of pile, as shown in Figure 10. Figure 12 shows that the deformation state of pile has been changed by the anchor. Based on the theory of structural mechanics, the pile without anchor is a cantilever structure with some location just under the sliding surface being fixed end, but the pile-anchor structure is a statically indeterminate cantilever beam with one end fixed and another end hinged at anchor head. Therefore, in the practical engineering, the direction of reinforcement in pile should be noted.

## 4. Conclusions

For the purpose of showing the load sharing ratio and distribution of landslide thrust in pile-anchor structure before failure in landslide, the physical scale model tests are conducted based on photoelastic technique. The effects of different material and pile length are investigated by 6 photoelastic test cases under different loading conditions. The conclusion can be highlighted as follows:

The load sharing ratio of anchor increased proportionally with a decrease of embed depth of pile and an increase of landslide thrust, and there is an obvious exponential function relation between the load sharing ratio of anchor and the landslide thrust;

The load sharing ratio of anchor shows an increasing trend but a slower and slower upward trend with the increase of landslide thrust, especially when the load sharing ratio of anchor is more than 20%; The load sharing ratio between anchor and pile would be finally expected to remain roughly stable;

When the embed depth of pile is small, the ratio of anchor tension in different models is closer to 1, showing that pile material has a lesser effect on bearing landslide thrust in this case. The proportion of landslide thrust is higher only in the case of larger embedding depth;

It is an injustice to blindly increase the pile length to reduce the internal force on pile. Compared with that method, the pile-anchor structure is significantly better at reducing bending moment value and optimizing bending moment distribution of pile.

This research is expected to improve understanding of the mechanism of the pile-anchor structure. As a result of the stress visualization, photoelastic technique would be the one of most potential research methods in geotechnical mechanical research field. In future studies, more detailed analysis on the complex correlation among the factors will be addressed by continuous measurements of the relationship among landslide thrust distribution, support structure and long evolution process of landslide.

## Figures and Tables

**Table 1:** Geometrical sizes of the pile in the model (Unit: mm).

No.	Pile Length	Embed Part of Pile	Cantilever Part of Pile
1	150	75	75
2	125	50	75
3	100	25	75

**Table 2:** Anchor tension and the ratio of loading share in epoxy resin pile model (Unit: N).

Landslide Thrust	No.1 Epoxy Resin Pile		No.2 Epoxy Resin Pile		No.3 Epoxy Resin Pile	
	Anchor Tension	Ratio of Loading Share	Anchor Tension	Ratio of Loading Share	Anchor Tension	Ratio of Loading Share
49	/	/	/	/	1.82	3.7%
98	8.51	8.7%	12.36	12.6%	18.72	19.1%
147	21.38	14.5%	25.23	17.1%	33.72	22.9%
196	35.78	18.2%	41.65	21.3%	49.62	25.3%
245	50.48	20.6%	57.84	23.6%	68.58	28.0%

**Table 3:** Anchor tension and the ratio of loading share in aluminum alloy pile model (Unit: N).

Landslide Thrust	No.1 Aluminum Alloy Pile		No.2 Aluminum Alloy Pile		No.3 Aluminum Alloy Pile	
	Anchor Tension	Ratio of Loading Share	Anchor Tension	Ratio of Loading Share	Anchor Tension	Ratio of Loading Share
49	/	/	/	/	/	/
98	/	/	7.84	8%	16.52	16.86%
147	3.43	2.33%	22.09	15%	31.06	21.13%
196	8.59	4.38%	34.28	17.49%	45.28	23.1%
245	16.52	6.74%	48.23	19.68%	59.38	24.2%
